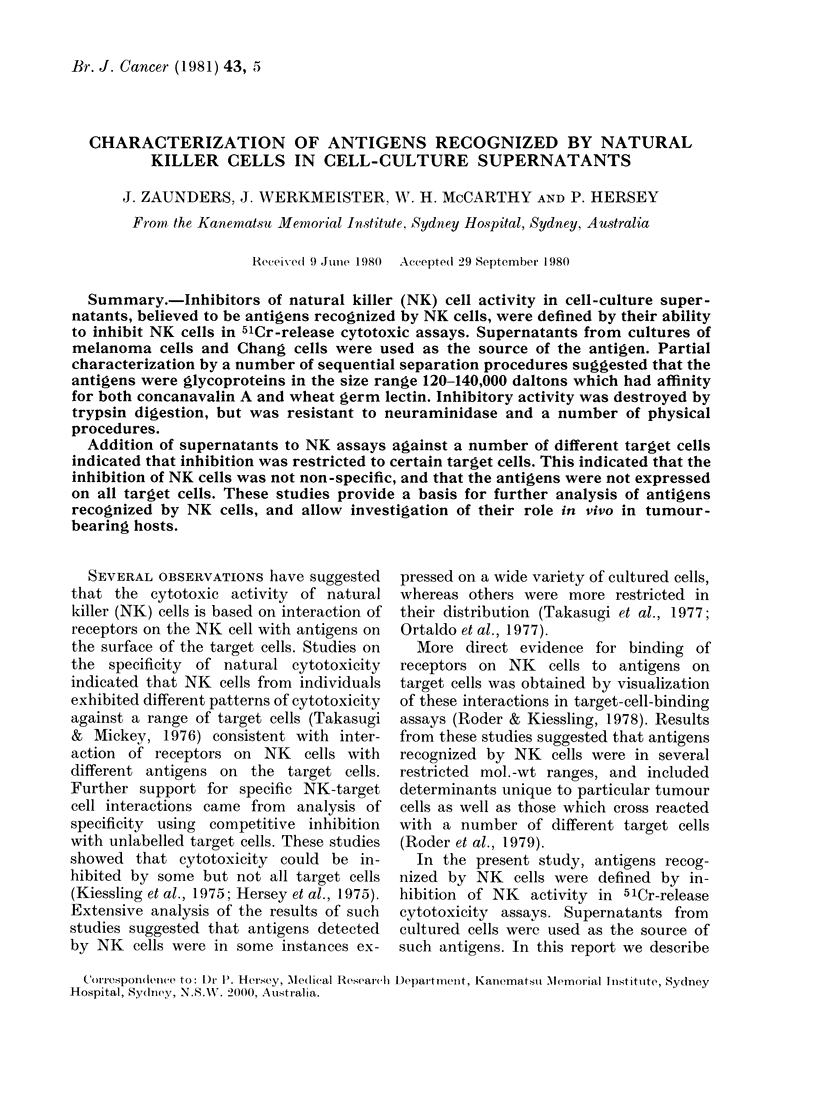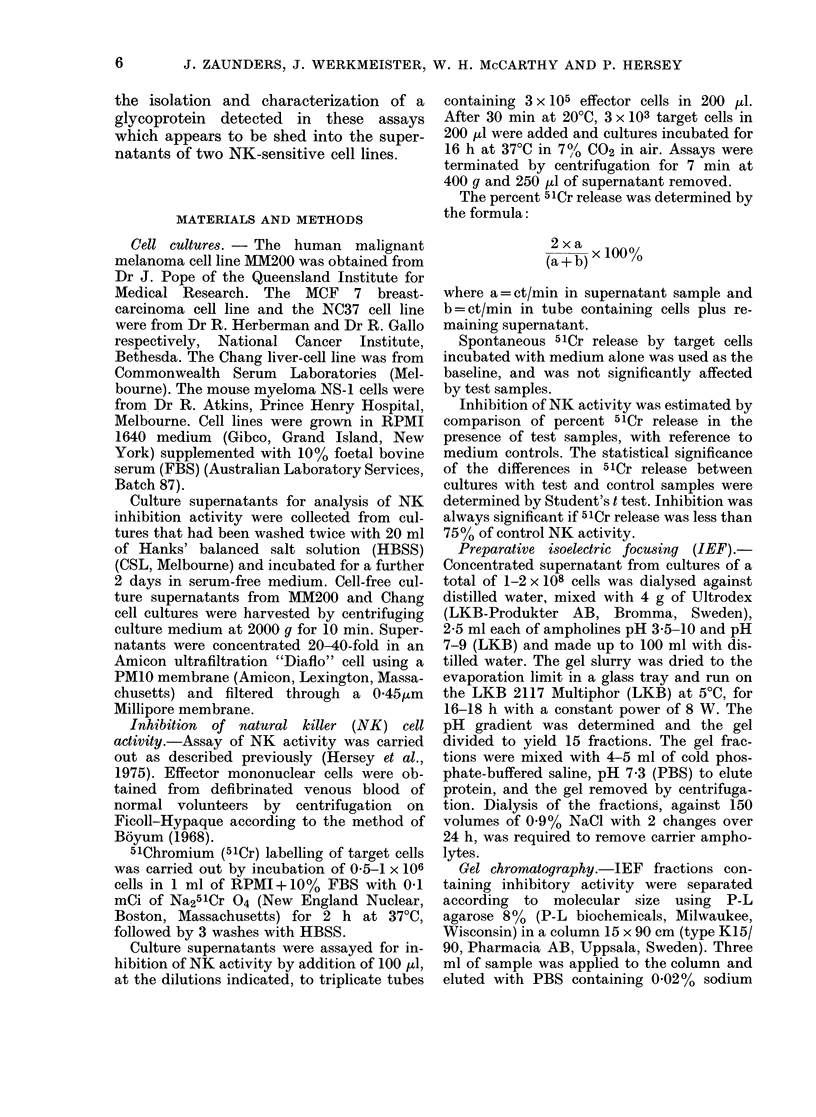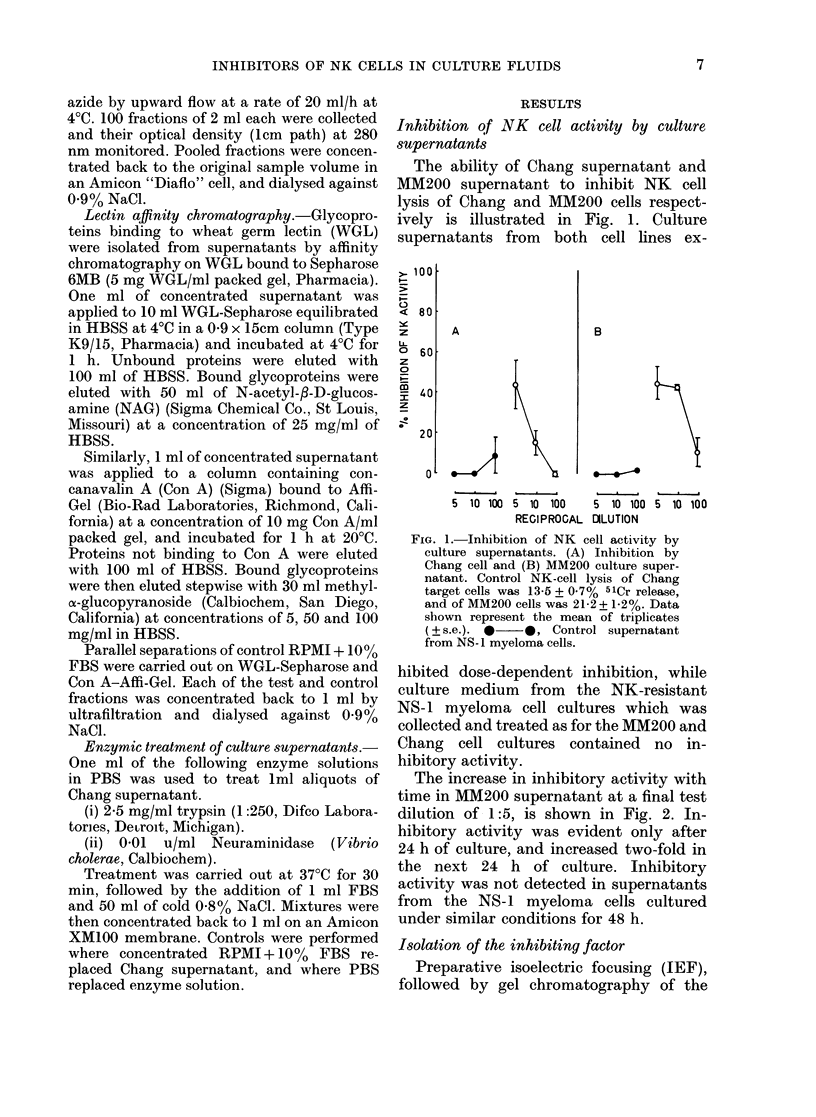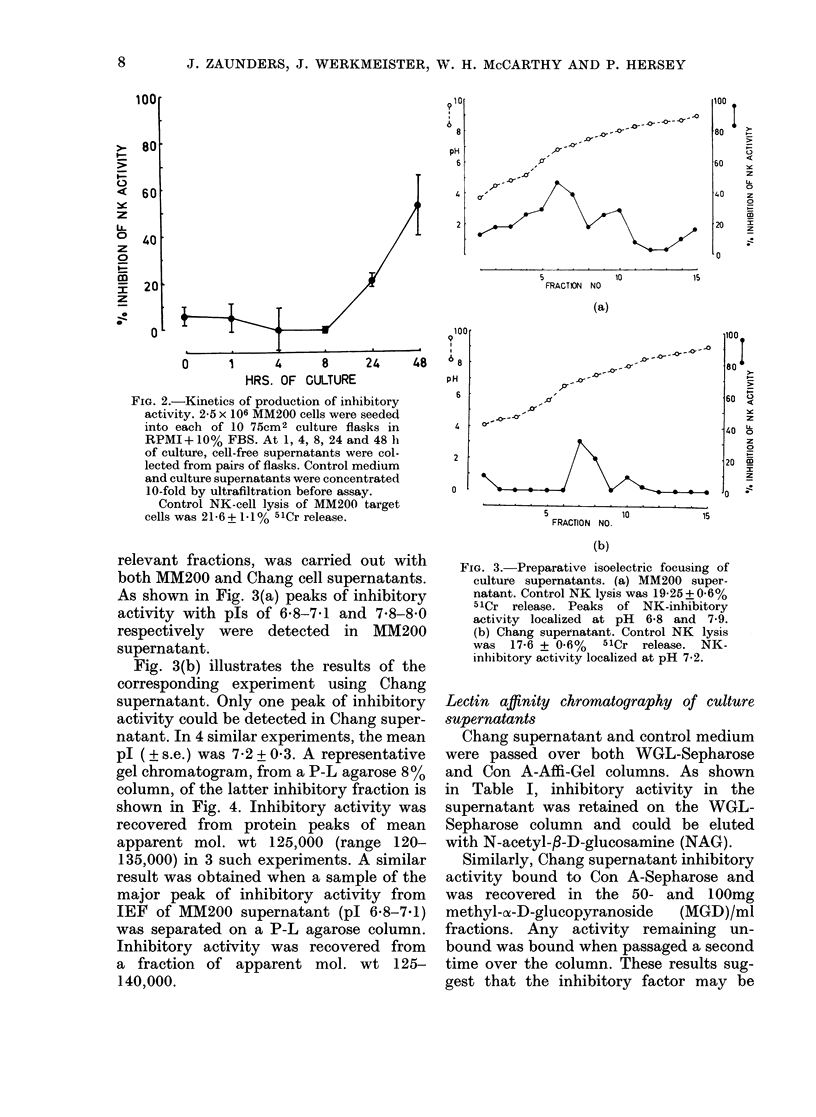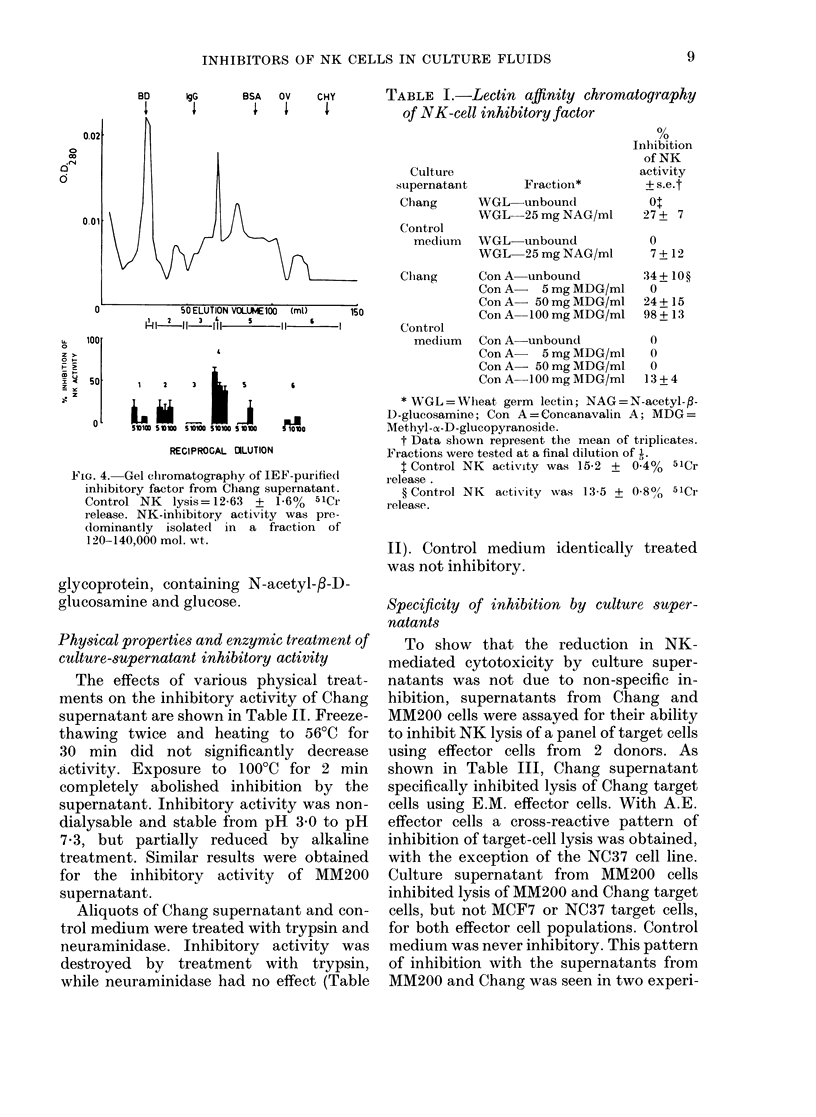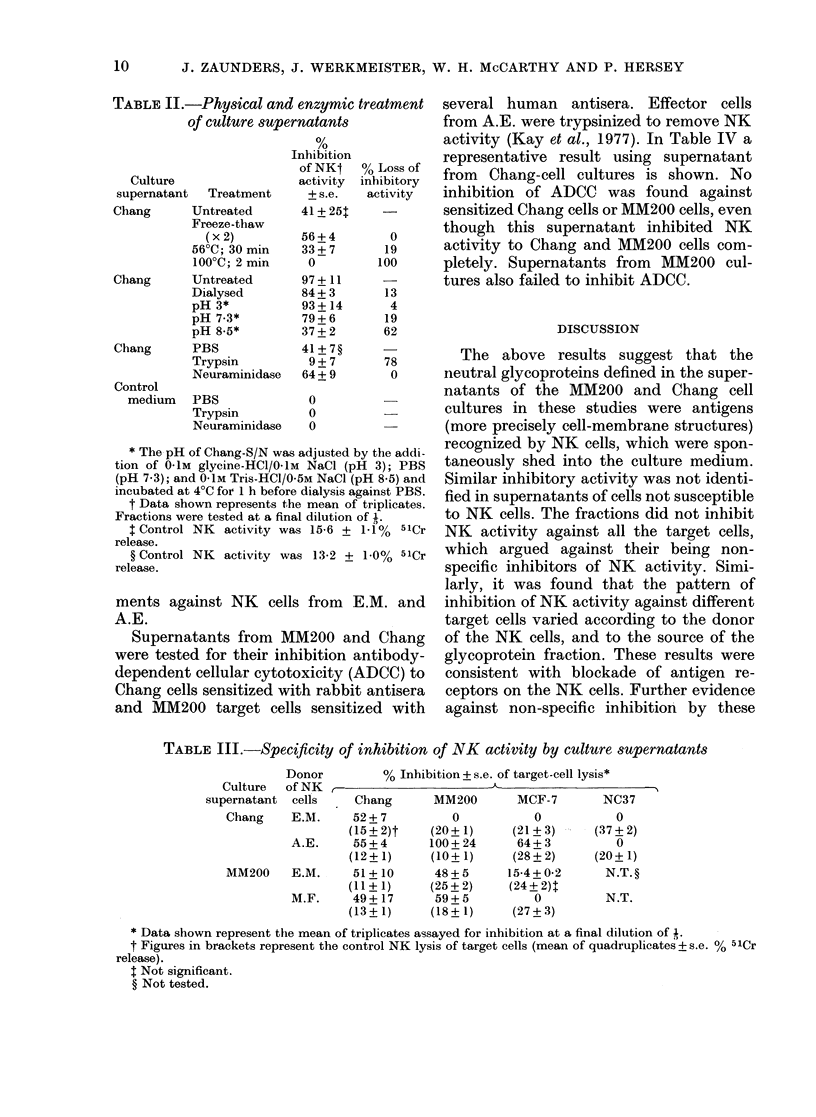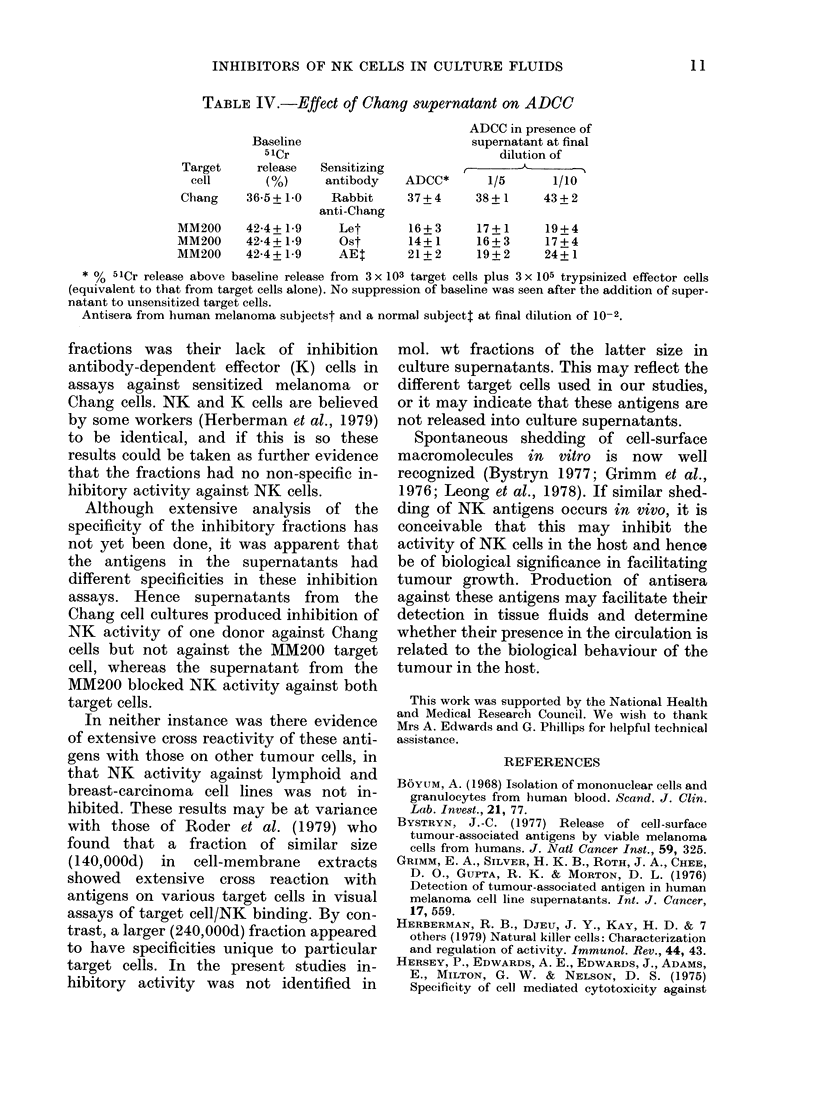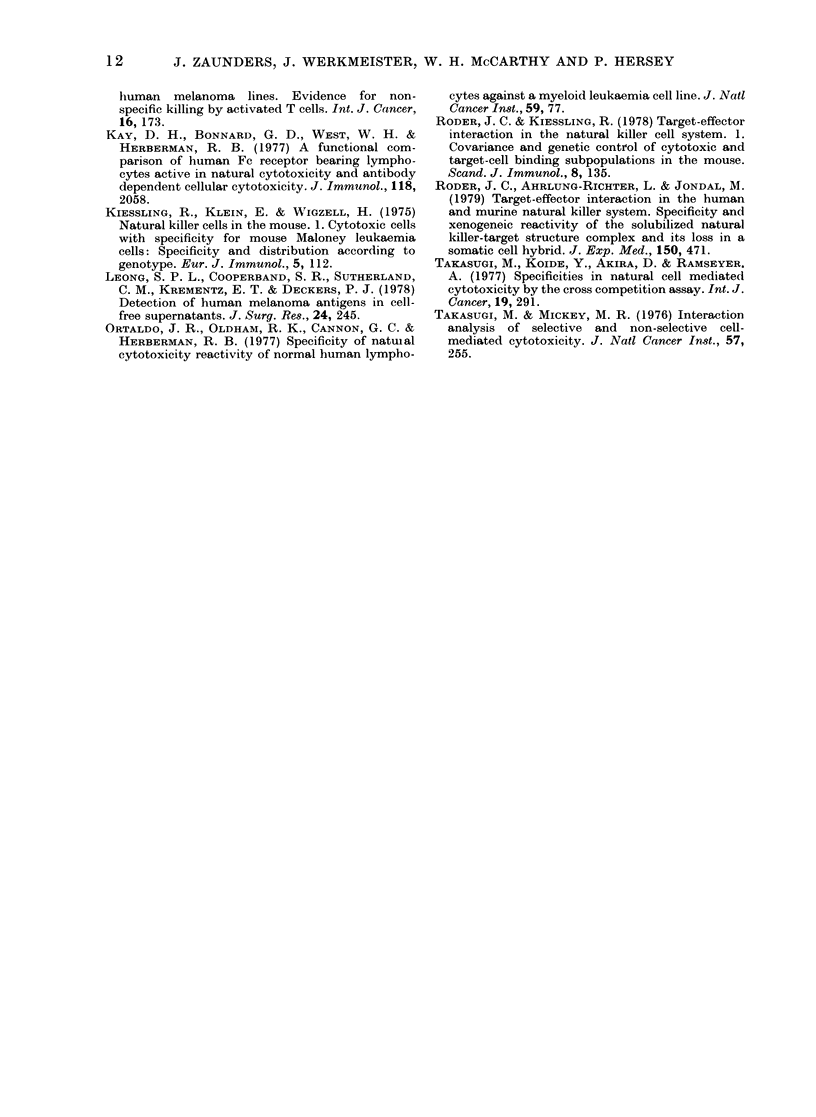# Characterization of antigens recognized by natural killer cells in cell-culture supernatants.

**DOI:** 10.1038/bjc.1981.2

**Published:** 1981-01

**Authors:** J. Zaunders, J. Werkmeister, W. H. McCarthy, P. Hersey

## Abstract

Inhibitors of natural killer (NK) cell activity in cell-culture supernatants, believed to be antigens recognized by NK cells, were defined by their ability to inhibit NK cells in 51Cr-release cytotoxic assays. Supernatants from cultures of melanoma cells and Chang cells were used as the source of the antigen. Partial characterization by a number of sequential separation procedures suggested that the antigens were glycoproteins in the size range 120-140,000 daltons which had affinity for both concanavalin A and wheat germ lectin. Inhibitory activity was destroyed by trypsin digestion, but was resistant to neuraminidase and a number of physical procedures. Addition of supernatants to NK assays against a number of different target cells indicated that inhibition was restricted to certain target cells. This indicated that the inhibition of NK cells was not non-specific, and that the antigens were not expressed on all target cells. These studies provide a basis for further analysis of antigens recognized by NK cells, and allow investigation of their role in vivo in tumour-bearing hosts.


*Br. J. Cancer* (1981) **43**, 5

### CHARACTERIZATION OF ANTIGENS RECOGNIZED BY NATURAL KILLER CELLS IN CELL-CULTURE SUPERNATANTS

J. ZAUNDERS, J. WERKMEISTER, W. H. McCARTHY AND P. HERSEY

*From the Kanematsu Memorial Institute, Sydney Hospital, Sydney, Australia*

Received 9 June 1980 Accepted 29 September 1980

**Summary.—Inhibitors of natural killer (NK) cell activity in cell-culture supernatants, believed to be antigens recognized by NK cells, were defined by their ability to inhibit NK cells in $^{51}$Cr-release cytotoxic assays. Supernatants from cultures of melanoma cells and Chang cells were used as the source of the antigen. Partial characterization by a number of sequential separation procedures suggested that the antigens were glycoproteins in the size range 120–140,000 daltons which had affinity for both concanavalin A and wheat germ lectin. Inhibitory activity was destroyed by trypsin digestion, but was resistant to neuraminidase and a number of physical procedures.**

**Addition of supernatants to NK assays against a number of different target cells indicated that inhibition was restricted to certain target cells. This indicated that the inhibition of NK cells was not non-specific, and that the antigens were not expressed on all target cells. These studies provide a basis for further analysis of antigens recognized by NK cells, and allow investigation of their role *in vivo* in tumour-bearing hosts.**

SEVERAL OBSERVATIONS have suggested that the cytotoxic activity of natural killer (NK) cells is based on interaction of receptors on the NK cell with antigens on the surface of the target cells. Studies on the specificity of natural cytotoxicity indicated that NK cells from individuals exhibited different patterns of cytotoxicity against a range of target cells (Takasugi & Mickey, 1976) consistent with interaction of receptors on NK cells with different antigens on the target cells. Further support for specific NK-target cell interactions came from analysis of specificity using competitive inhibition with unlabelled target cells. These studies showed that cytotoxicity could be inhibited by some but not all target cells (Kiessling *et al.*, 1975; Hersey *et al.*, 1975). Extensive analysis of the results of such studies suggested that antigens detected by NK cells were in some instances expressed on a wide variety of cultured cells, whereas others were more restricted in their distribution (Takasugi *et al.*, 1977; Ortaldo *et al.*, 1977).

More direct evidence for binding of receptors on NK cells to antigens on target cells was obtained by visualization of these interactions in target-cell-binding assays (Roder & Kiessling, 1978). Results from these studies suggested that antigens recognized by NK cells were in several restricted mol.-wt ranges, and included determinants unique to particular tumour cells as well as those which cross reacted with a number of different target cells (Roder *et al.*, 1979).

In the present study, antigens recognized by NK cells were defined by inhibition of NK activity in $^{51}$Cr-release cytotoxicity assays. Supernatants from cultured cells were used as the source of such antigens. In this report we describe

Correspondence to: Dr P. Hersey, Medical Research Department, Kanematsu Memorial Institute, Sydney Hospital, Sydney, N.S.W. 2000, Australia.

the isolation and characterization of a glycoprotein detected in these assays which appears to be shed into the supernatants of two NK-sensitive cell lines.

### MATERIALS AND METHODS

*Cell cultures.* — The human malignant melanoma cell line MM200 was obtained from Dr J. Pope of the Queensland Institute for Medical Research. The MCF 7 breast-carcinoma cell line and the NC37 cell line were from Dr R. Herberman and Dr R. Gallo respectively, National Cancer Institute, Bethesda. The Chang liver-cell line was from Commonwealth Serum Laboratories (Melbourne). The mouse myeloma NS-1 cells were from Dr R. Atkins, Prince Henry Hospital, Melbourne. Cell lines were grown in RPMI 1640 medium (Gibco, Grand Island, New York) supplemented with 10% foetal bovine serum (FBS) (Australian Laboratory Services, Batch 87).

Culture supernatants for analysis of NK inhibition activity were collected from cultures that had been washed twice with 20 ml of Hanks' balanced salt solution (HBSS) (CSL, Melbourne) and incubated for a further 2 days in serum-free medium. Cell-free culture supernatants from MM200 and Chang cell cultures were harvested by centrifuging culture medium at 2000 *g* for 10 min. Supernatants were concentrated 20–40-fold in an Amicon ultrafiltration "Diaflo" cell using a PM10 membrane (Amicon, Lexington, Massachusetts) and filtered through a 0·45$\mu$m Millipore membrane.

*Inhibition of natural killer* (*NK*) *cell activity.*—Assay of NK activity was carried out as described previously (Hersey *et al.*, 1975). Effector mononuclear cells were obtained from defibrinated venous blood of normal volunteers by centrifugation on Ficoll–Hypaque according to the method of Böyum (1968).

$^{51}$Chromium ($^{51}$Cr) labelling of target cells was carried out by incubation of 0·5–1 $\times 10^6$ cells in 1 ml of RPMI + 10% FBS with 0·1 mCi of $Na_2{}^{51}Cr\ O_4$ (New England Nuclear, Boston, Massachusetts) for 2 h at 37°C, followed by 3 washes with HBSS.

Culture supernatants were assayed for inhibition of NK activity by addition of 100 $\mu$l, at the dilutions indicated, to triplicate tubes containing $3 \times 10^5$ effector cells in 200 $\mu$l. After 30 min at 20°C, $3 \times 10^3$ target cells in 200 $\mu$l were added and cultures incubated for 16 h at 37°C in 7% $CO_2$ in air. Assays were terminated by centrifugation for 7 min at 400 *g* and 250 $\mu$l of supernatant removed.

The percent $^{51}$Cr release was determined by the formula:

$$\frac{2 \times a}{(a + b)} \times 100\%$$

where a = ct/min in supernatant sample and b = ct/min in tube containing cells plus remaining supernatant.

Spontaneous $^{51}$Cr release by target cells incubated with medium alone was used as the baseline, and was not significantly affected by test samples.

Inhibition of NK activity was estimated by comparison of percent $^{51}$Cr release in the presence of test samples, with reference to medium controls. The statistical significance of the differences in $^{51}$Cr release between cultures with test and control samples were determined by Student's *t* test. Inhibition was always significant if $^{51}$Cr release was less than 75% of control NK activity.

*Preparative isoelectric focusing* (*IEF*).—Concentrated supernatant from cultures of a total of 1–2 $\times 10^8$ cells was dialysed against distilled water, mixed with 4 g of Ultrodex (LKB-Produkter AB, Bromma, Sweden), 2·5 ml each of ampholines pH 3·5–10 and pH 7–9 (LKB) and made up to 100 ml with distilled water. The gel slurry was dried to the evaporation limit in a glass tray and run on the LKB 2117 Multiphor (LKB) at 5°C, for 16–18 h with a constant power of 8 W. The pH gradient was determined and the gel divided to yield 15 fractions. The gel fractions were mixed with 4–5 ml of cold phosphate-buffered saline, pH 7·3 (PBS) to elute protein, and the gel removed by centrifugation. Dialysis of the fractions, against 150 volumes of 0·9% NaCl with 2 changes over 24 h, was required to remove carrier ampholytes.

*Gel chromatography.*—IEF fractions containing inhibitory activity were separated according to molecular size using P-L agarose 8% (P-L biochemicals, Milwaukee, Wisconsin) in a column 15 × 90 cm (type K15/90, Pharmacia AB, Uppsala, Sweden). Three ml of sample was applied to the column and eluted with PBS containing 0·02% sodium azide by upward flow at a rate of 20 ml/h at 4°C. 100 fractions of 2 ml each were collected and their optical density (1cm path) at 280 nm monitored. Pooled fractions were concentrated back to the original sample volume in an Amicon "Diaflo" cell, and dialysed against 0·9% NaCl.

*Lectin affinity chromatography.*—Glycoproteins binding to wheat germ lectin (WGL) were isolated from supernatants by affinity chromatography on WGL bound to Sepharose 6MB (5 mg WGL/ml packed gel, Pharmacia). One ml of concentrated supernatant was applied to 10 ml WGL-Sepharose equilibrated in HBSS at 4°C in a 0·9 x 15cm column (Type K9/15, Pharmacia) and incubated at 4°C for 1 h. Unbound proteins were eluted with 100 ml of HBSS. Bound glycoproteins were eluted with 50 ml of N-acetyl-$\beta$-D-glucosamine (NAG) (Sigma Chemical Co., St Louis, Missouri) at a concentration of 25 mg/ml of HBSS.

Similarly, 1 ml of concentrated supernatant was applied to a column containing concanavalin A (Con A) (Sigma) bound to Affi-Gel (Bio-Rad Laboratories, Richmond, California) at a concentration of 10 mg Con A/ml packed gel, and incubated for 1 h at 20°C. Proteins not binding to Con A were eluted with 100 ml of HBSS. Bound glycoproteins were then eluted stepwise with 30 ml methyl-$\alpha$-glucopyranoside (Calbiochem, San Diego, California) at concentrations of 5, 50 and 100 mg/ml in HBSS.

Parallel separations of control RPMI + 10% FBS were carried out on WGL-Sepharose and Con A–Affi-Gel. Each of the test and control fractions was concentrated back to 1 ml by ultrafiltration and dialysed against 0·9% NaCl.

*Enzymic treatment of culture supernatants.*—One ml of the following enzyme solutions in PBS was used to treat 1ml aliquots of Chang supernatant.

(i) 2·5 mg/ml trypsin (1:250, Difco Laboratories, Detroit, Michigan).

(ii) 0·01 u/ml Neuraminidase (*Vibrio cholerae*, Calbiochem).

Treatment was carried out at 37°C for 30 min, followed by the addition of 1 ml FBS and 50 ml of cold 0·8% NaCl. Mixtures were then concentrated back to 1 ml on an Amicon XM100 membrane. Controls were performed where concentrated RPMI + 10% FBS replaced Chang supernatant, and where PBS replaced enzyme solution.

### RESULTS

### *Inhibition of NK cell activity by culture supernatants*

The ability of Chang supernatant and MM200 supernatant to inhibit NK cell lysis of Chang and MM200 cells respectively is illustrated in Fig. 1. Culture supernatants from both cell lines ex-

FIG. 1.—Inhibition of NK cell activity by culture supernatants. (A) Inhibition by Chang cell and (B) MM200 culture supernatant. Control NK-cell lysis of Chang target cells was 13·5 ± 0·7% $^{51}$Cr release, and of MM200 cells was 21·2 ± 1·2%. Data shown represent the mean of triplicates (± s.e.). ●——●, Control supernatant from NS-1 myeloma cells.

hibited dose-dependent inhibition, while culture medium from the NK-resistant NS-1 myeloma cell cultures which was collected and treated as for the MM200 and Chang cell cultures contained no inhibitory activity.

The increase in inhibitory activity with time in MM200 supernatant at a final test dilution of 1:5, is shown in Fig. 2. Inhibitory activity was evident only after 24 h of culture, and increased two-fold in the next 24 h of culture. Inhibitory activity was not detected in supernatants from the NS-1 myeloma cells cultured under similar conditions for 48 h.

### *Isolation of the inhibiting factor*

Preparative isoelectric focusing (IEF), followed by gel chromatography of the

FIG. 2.—Kinetics of production of inhibitory activity. $2 \cdot 5 \times 10^6$ MM200 cells were seeded into each of 10 $75cm^2$ culture flasks in RPMI + 10% FBS. At 1, 4, 8, 24 and 48 h of culture, cell-free supernatants were collected from pairs of flasks. Control medium and culture supernatants were concentrated 10-fold by ultrafiltration before assay. Control NK-cell lysis of MM200 target cells was $21 \cdot 6 \pm 1 \cdot 1$% $^{51}Cr$ release.

FIG. 3.—Preparative isoelectric focusing of culture supernatants. (a) MM200 supernatant. Control NK lysis was $19 \cdot 25 \pm 0 \cdot 6$% $^{51}Cr$ release. Peaks of NK-inhibitory activity localized at pH 6·8 and 7·9. (b) Chang supernatant. Control NK lysis was $17 \cdot 6 \pm 0 \cdot 6$% $^{51}Cr$ release. NK-inhibitory activity localized at pH 7·2.

relevant fractions, was carried out with both MM200 and Chang cell supernatants. As shown in Fig. 3(a) peaks of inhibitory activity with pIs of 6·8–7·1 and 7·8–8·0 respectively were detected in MM200 supernatant.

Fig. 3(b) illustrates the results of the corresponding experiment using Chang supernatant. Only one peak of inhibitory activity could be detected in Chang supernatant. In 4 similar experiments, the mean pI ($\pm$s.e.) was $7 \cdot 2 \pm 0 \cdot 3$. A representative gel chromatogram, from a P-L agarose 8% column, of the latter inhibitory fraction is shown in Fig. 4. Inhibitory activity was recovered from protein peaks of mean apparent mol. wt 125,000 (range 120–135,000) in 3 such experiments. A similar result was obtained when a sample of the major peak of inhibitory activity from IEF of MM200 supernatant (pI 6·8–7·1) was separated on a P-L agarose column. Inhibitory activity was recovered from a fraction of apparent mol. wt 125–140,000.

*Lectin affinity chromatography of culture supernatants*

Chang supernatant and control medium were passed over both WGL-Sepharose and Con A-Affi-Gel columns. As shown in Table I, inhibitory activity in the supernatant was retained on the WGL-Sepharose column and could be eluted with N-acetyl-β-D-glucosamine (NAG).

Similarly, Chang supernatant inhibitory activity bound to Con A-Sepharose and was recovered in the 50- and 100mg methyl-α-D-glucopyranoside (MGD)/ml fractions. Any activity remaining unbound was bound when passaged a second time over the column. These results suggest that the inhibitory factor may be

FIG. 4.—Gel chromatography of IEF-purified inhibitory factor from Chang supernatant. Control NK lysis = 12·63 ± 1·6% $^{51}Cr$ release. NK-inhibitory activity was predominantly isolated in a fraction of 120–140,000 mol. wt.

glycoprotein, containing N-acetyl-β-D-glucosamine and glucose.

*Physical properties and enzymic treatment of culture-supernatant inhibitory activity*

The effects of various physical treatments on the inhibitory activity of Chang supernatant are shown in Table II. Freeze-thawing twice and heating to 56°C for 30 min did not significantly decrease activity. Exposure to 100°C for 2 min completely abolished inhibition by the supernatant. Inhibitory activity was non-dialysable and stable from pH 3·0 to pH 7·3, but partially reduced by alkaline treatment. Similar results were obtained for the inhibitory activity of MM200 supernatant.

Aliquots of Chang supernatant and control medium were treated with trypsin and neuraminidase. Inhibitory activity was destroyed by treatment with trypsin, while neuraminidase had no effect (Table

TABLE I.—*Lectin affinity chromatography of NK-cell inhibitory factor*

| Culture supernatant | Fraction* | % Inhibition of NK activity ± s.e.† |
|---|---|---|
| Chang | WGL—unbound | 0‡ |
| | WGL—25 mg NAG/ml | 27 ± 7 |
| Control medium | WGL—unbound | 0 |
| | WGL—25 mg NAG/ml | 7 ± 12 |
| Chang | Con A—unbound | 34 ± 10§ |
| | Con A— 5 mg MDG/ml | 0 |
| | Con A— 50 mg MDG/ml | 24 ± 15 |
| | Con A—100 mg MDG/ml | 98 ± 13 |
| Control medium | Con A—unbound | 0 |
| | Con A— 5 mg MDG/ml | 0 |
| | Con A— 50 mg MDG/ml | 0 |
| | Con A—100 mg MDG/ml | 13 ± 4 |

* WGL = Wheat germ lectin; NAG = N-acetyl-β-D-glucosamine; Con A = Concanavalin A; MDG = Methyl-α-D-glucopyranoside.

† Data shown represent the mean of triplicates. Fractions were tested at a final dilution of $\frac{1}{5}$.

‡ Control NK activity was 15·2 ± 0·4% $^{51}Cr$ release .

§ Control NK activity was 13·5 ± 0·8% $^{51}Cr$ release.

II). Control medium identically treated was not inhibitory.

*Specificity of inhibition by culture supernatants*

To show that the reduction in NK-mediated cytotoxicity by culture supernatants was not due to non-specific inhibition, supernatants from Chang and MM200 cells were assayed for their ability to inhibit NK lysis of a panel of target cells using effector cells from 2 donors. As shown in Table III, Chang supernatant specifically inhibited lysis of Chang target cells using E.M. effector cells. With A.E. effector cells a cross-reactive pattern of inhibition of target-cell lysis was obtained, with the exception of the NC37 cell line. Culture supernatant from MM200 cells inhibited lysis of MM200 and Chang target cells, but not MCF7 or NC37 target cells, for both effector cell populations. Control medium was never inhibitory. This pattern of inhibition with the supernatants from MM200 and Chang was seen in two experi-

TABLE II.—*Physical and enzymic treatment of culture supernatants*

| Culture supernatant | Treatment | % Inhibition of NK† activity ± s.e. | % Loss of inhibitory activity |
|---|---|---|---|
| Chang | Untreated | 41 ± 25‡ | — |
| | Freeze-thaw (× 2) | 56 ± 4 | 0 |
| | 56°C; 30 min | 33 ± 7 | 19 |
| | 100°C; 2 min | 0 | 100 |
| Chang | Untreated | 97 ± 11 | — |
| | Dialysed | 84 ± 3 | 13 |
| | pH 3* | 93 ± 14 | 4 |
| | pH 7·3* | 79 ± 6 | 19 |
| | pH 8·5* | 37 ± 2 | 62 |
| Chang | PBS | 41 ± 7§ | — |
| | Trypsin | 9 ± 7 | 78 |
| | Neuraminidase | 64 ± 9 | 0 |
| Control medium | PBS | 0 | — |
| | Trypsin | 0 | — |
| | Neuraminidase | 0 | — |

* The pH of Chang-S/N was adjusted by the addition of 0·1M glycine-HCl/0·1M NaCl (pH 3); PBS (pH 7·3); and 0·1M Tris-HCl/0·5M NaCl (pH 8·5) and incubated at 4°C for 1 h before dialysis against PBS.

† Data shown represents the mean of triplicates. Fractions were tested at a final dilution of $\frac{1}{5}$.

‡ Control NK activity was 15·6 ± 1·1% $^{51}$Cr release.

§ Control NK activity was 13·2 ± 1·0% $^{51}$Cr release.

ments against NK cells from E.M. and A.E.

Supernatants from MM200 and Chang were tested for their inhibition antibody-dependent cellular cytotoxicity (ADCC) to Chang cells sensitized with rabbit antisera and MM200 target cells sensitized with several human antisera. Effector cells from A.E. were trypsinized to remove NK activity (Kay *et al.*, 1977). In Table IV a representative result using supernatant from Chang-cell cultures is shown. No inhibition of ADCC was found against sensitized Chang cells or MM200 cells, even though this supernatant inhibited NK activity to Chang and MM200 cells completely. Supernatants from MM200 cultures also failed to inhibit ADCC.

### DISCUSSION

The above results suggest that the neutral glycoproteins defined in the supernatants of the MM200 and Chang cell cultures in these studies were antigens (more precisely cell-membrane structures) recognized by NK cells, which were spontaneously shed into the culture medium. Similar inhibitory activity was not identified in supernatants of cells not susceptible to NK cells. The fractions did not inhibit NK activity against all the target cells, which argued against their being non-specific inhibitors of NK activity. Similarly, it was found that the pattern of inhibition of NK activity against different target cells varied according to the donor of the NK cells, and to the source of the glycoprotein fraction. These results were consistent with blockade of antigen receptors on the NK cells. Further evidence against non-specific inhibition by these

TABLE III.—*Specificity of inhibition of NK activity by culture supernatants*

| Culture supernatant | Donor of NK cells | % Inhibition ± s.e. of target-cell lysis* | | | |
|---|---|---|---|---|---|
| | | Chang | MM200 | MCF-7 | NC37 |
| Chang | E.M. | 52 ± 7 | 0 | 0 | 0 |
| | | (15 ± 2)† | (20 ± 1) | (21 ± 3) | (37 ± 2) |
| | A.E. | 55 ± 4 | 100 ± 24 | 64 ± 3 | 0 |
| | | (12 ± 1) | (10 ± 1) | (28 ± 2) | (20 ± 1) |
| MM200 | E.M. | 51 ± 10 | 48 ± 5 | 15·4 ± 0·2 | N.T.§ |
| | | (11 ± 1) | (25 ± 2) | (24 ± 2)‡ | |
| | M.F. | 49 ± 17 | 59 ± 5 | 0 | N.T. |
| | | (13 ± 1) | (18 ± 1) | (27 ± 3) | |

* Data shown represent the mean of triplicates assayed for inhibition at a final dilution of $\frac{1}{5}$.

† Figures in brackets represent the control NK lysis of target cells (mean of quadruplicates ± s.e. % $^{51}$Cr release).

‡ Not significant.

§ Not tested.

TABLE IV.—*Effect of Chang supernatant on ADCC*

| Target cell | Baseline $^{51}Cr$ release (%) | Sensitizing antibody | ADCC* | ADCC in presence of supernatant at final dilution of 1/5 | 1/10 |
|---|---|---|---|---|---|
| Chang | 36·5 ± 1·0 | Rabbit anti-Chang | 37 ± 4 | 38 ± 1 | 43 ± 2 |
| MM200 | 42·4 ± 1·9 | Le† | 16 ± 3 | 17 ± 1 | 19 ± 4 |
| MM200 | 42·4 ± 1·9 | Os† | 14 ± 1 | 16 ± 3 | 17 ± 4 |
| MM200 | 42·4 ± 1·9 | AE‡ | 21 ± 2 | 19 ± 2 | 24 ± 1 |

* % $^{51}Cr$ release above baseline release from $3 \times 10^3$ target cells plus $3 \times 10^5$ trypsinized effector cells (equivalent to that from target cells alone). No suppression of baseline was seen after the addition of supernatant to unsensitized target cells.

Antisera from human melanoma subjects† and a normal subject‡ at final dilution of $10^{-2}$.

fractions was their lack of inhibition antibody-dependent effector (K) cells in assays against sensitized melanoma or Chang cells. NK and K cells are believed by some workers (Herberman *et al.*, 1979) to be identical, and if this is so these results could be taken as further evidence that the fractions had no non-specific inhibitory activity against NK cells.

Although extensive analysis of the specificity of the inhibitory fractions has not yet been done, it was apparent that the antigens in the supernatants had different specificities in these inhibition assays. Hence supernatants from the Chang cell cultures produced inhibition of NK activity of one donor against Chang cells but not against the MM200 target cell, whereas the supernatant from the MM200 blocked NK activity against both target cells.

In neither instance was there evidence of extensive cross reactivity of these antigens with those on other tumour cells, in that NK activity against lymphoid and breast-carcinoma cell lines was not inhibited. These results may be at variance with those of Roder *et al.* (1979) who found that a fraction of similar size (140,000d) in cell-membrane extracts showed extensive cross reaction with antigens on various target cells in visual assays of target cell/NK binding. By contrast, a larger (240,000d) fraction appeared to have specificities unique to particular target cells. In the present studies inhibitory activity was not identified in mol. wt fractions of the latter size in culture supernatants. This may reflect the different target cells used in our studies, or it may indicate that these antigens are not released into culture supernatants.

Spontaneous shedding of cell-surface macromolecules *in vitro* is now well recognized (Bystryn 1977; Grimm *et al.*, 1976; Leong *et al.*, 1978). If similar shedding of NK antigens occurs *in vivo*, it is conceivable that this may inhibit the activity of NK cells in the host and hence be of biological significance in facilitating tumour growth. Production of antisera against these antigens may facilitate their detection in tissue fluids and determine whether their presence in the circulation is related to the biological behaviour of the tumour in the host.

This work was supported by the National Health and Medical Research Council. We wish to thank Mrs A. Edwards and G. Phillips for helpful technical assistance.

REFERENCES

BÖYUM, A. (1968) Isolation of mononuclear cells and granulocytes from human blood. *Scand. J. Clin. Lab. Invest.*, **21**, 77.

BYSTRYN, J.-C. (1977) Release of cell-surface tumour-associated antigens by viable melanoma cells from humans. *J. Natl Cancer Inst.*, **59**, 325.

GRIMM, E. A., SILVER, H. K. B., ROTH, J. A., CHEE, D. O., GUPTA, R. K. & MORTON, D. L. (1976) Detection of tumour-associated antigen in human melanoma cell line supernatants. *Int. J. Cancer*, **17**, 559.

HERBERMAN, R. B., DJEU, J. Y., KAY, H. D. & 7 others (1979) Natural killer cells: Characterization and regulation of activity. *Immunol. Rev.*, **44**, 43.

HERSEY, P., EDWARDS, A. E., EDWARDS, J., ADAMS, E., MILTON, G. W. & NELSON, D. S. (1975) Specificity of cell mediated cytotoxicity against human melanoma lines. Evidence for non-specific killing by activated T cells. *Int. J. Cancer*, **16**, 173.

KAY, D. H., BONNARD, G. D., WEST, W. H. & HERBERMAN, R. B. (1977) A functional comparison of human Fc receptor bearing lymphocytes active in natural cytotoxicity and antibody dependent cellular cytotoxicity. *J. Immunol.*, **118**, 2058.

KIESSLING, R., KLEIN, E. & WIGZELL, H. (1975) Natural killer cells in the mouse. 1. Cytotoxic cells with specificity for mouse Maloney leukaemia cells: Specificity and distribution according to genotype. *Eur. J. Immunol.*, **5**, 112.

LEONG, S. P. L., COOPERBAND, S. R., SUTHERLAND, C. M., KREMENTZ, E. T. & DECKERS, P. J. (1978) Detection of human melanoma antigens in cell-free supernatants. *J. Surg. Res.*, **24**, 245.

ORTALDO, J. R., OLDHAM, R. K., CANNON, G. C. & HERBERMAN, R. B. (1977) Specificity of natuial cytotoxicity reactivity of normal human lymphocytes against a myeloid leukaemia cell line. *J. Natl Cancer Inst.*, **59**, 77.

RODER, J. C. & KIESSLING, R. (1978) Target-effector interaction in the natural killer cell system. 1. Covariance and genetic control of cytotoxic and target-cell binding subpopulations in the mouse. *Scand. J. Immunol.*, **8**, 135.

RODER, J. C., AHRLUNG-RICHTER, L. & JONDAL, M. (1979) Target-effector interaction in the human and murine natural killer system. Specificity and xenogeneic reactivity of the solubilized natural killer-target structure complex and its loss in a somatic cell hybrid. *J. Exp. Med.*, **150**, 471.

TAKASUGI, M., KOIDE, Y., AKIRA, D. & RAMSEYER. A. (1977) Specificities in natural cell mediated cytotoxicity by the cross competition assay. *Int. J. Cancer*, **19**, 291.

TAKASUGI, M. & MICKEY, M. R. (1976) Interaction analysis of selective and non-selective cell-mediated cytotoxicity. *J. Natl Cancer Inst.*, **57**, 255.